# Reliable and stable fundus image registration based on brain-inspired spatially-varying adaptive pyramid context aggregation network

**DOI:** 10.3389/fnins.2022.1117134

**Published:** 2023-01-16

**Authors:** Jie Xu, Kang Yang, Youxin Chen, Liming Dai, Dongdong Zhang, Ping Shuai, Rongjie Shi, Zhanbo Yang

**Affiliations:** ^1^Beijing Institute of Ophthalmology, Beijing Tongren Eye Center, Beijing Tongren Hospital, Capital Medical University, Beijing Key Laboratory of Ophthalmology and Visual Sciences, Beijing, China; ^2^Beijing Zhizhen Internet Technology Co. Ltd., Beijing, China; ^3^Department of Ophthalmology, Peking Union Medical College Hospital, Beijing, China; ^4^Department of Health Management and Physical Examination, Sichuan Provincial People's Hospital, University of Electronic Science and Technology of China, Chengdu, China; ^5^School of Medicine, University of Electronic Science and Technology of China, Chengdu, China

**Keywords:** retinal image analysis, fundus image registration, deep learning, context aggregation, structured triplet ranking loss

## Abstract

The task of fundus image registration aims to find matching keypoints between an image pair. Traditional methods detect the keypoint by hand-designed features, which fail to cope with complex application scenarios. Due to the strong feature learning ability of deep neural network, current image registration methods based on deep learning directly learn to align the geometric transformation between the reference image and test image in an end-to-end manner. Another mainstream of this task aims to learn the displacement vector field between the image pair. In this way, the image registration has achieved significant advances. However, due to the complicated vascular morphology of retinal image, such as texture and shape, current widely used image registration methods based on deep learning fail to achieve reliable and stable keypoint detection and registration results. To this end, in this paper, we aim to bridge this gap. Concretely, since the vessel crossing and branching points can reliably and stably characterize the key components of fundus image, we propose to learn to detect and match all the crossing and branching points of the input images based on a single deep neural network. Moreover, in order to accurately locate the keypoints and learn discriminative feature embedding, a brain-inspired spatially-varying adaptive pyramid context aggregation network is proposed to incorporate the contextual cues under the supervision of structured triplet ranking loss. Experimental results show that the proposed method achieves more accurate registration results with significant speed advantage.

## 1. Introduction

Fundus image analysis has been widely researched, due to its significant advantage of non-invasive observation. The purpose of image registration (Hill et al., 2001; Sotiras et al., 2013) is to deform the test image to the coordinate system of the reference image, so that the same point can be imaged at the same coordinate of the two images (Oliveira and Tavares, 2014). Registration of medical images is a crucial step in the image processing. Image registration can trace the progression of the same patient through time, providing a basis for clinical diagnosis, lowering physician effort, and aiding in the investigation of disease prognosis and outcome. In order to accurately learn the deformation coefficient to transform the test image, the matching keypoints between the test image and reference image should be obtained. To this end, previous methods rely on human-designed features to distinguish among visually similar keypoints, by encoding the texture, shape or intensity gradient with particularly designed computing pattern. Recently, deep neural network (DNN) (Krizhevsky et al., 2012; Simonyan and Zisserman, 2014; He et al., 2016) based image registration has made rapid progress due to its strong feature learning ability. Some current DNN based methods propose to directly learn the geometric transformation, such as homography transformation, between the test image and reference image. Other works also aim to learn the dense pixel-level displacement vector filed between the image pair (Cao et al., 2017; Krebs et al., 2017). However, due to the complex and variable retinal vascular structure, previous methods fail to achieve **reliable** and **stable** registration performance, which severely limits downstream applications. Considering that the vessel crossing and branching points are able to reliably and stably characterize the fundus image (Deng et al., 2010; Chen et al., 2011), we propose to choose all the crossing and branching points as the keypoints. To this end, a single deep neural network is utilized to learn to simultaneously locate and match all the keypoints.

Since the lower-level spatial details and higher-level semantic cues of fundus image are both critical for learning accurate keypoint detection and corresponding discriminative feature embedding for keypoint matching, we employ the widely used encoder-decoder architecture (Ronneberger et al., 2015) as the basic network. Moreover, due to the large intra-class variability and small inter-class difference of fundus image, the non-matching keypoints are prone to be misclassified. It is natural for human being to gain the knowledge of contextual consistency, which is helpful for alleviating this issue. As a result, contextual cues should be incorporated into the vanilla encoder-decoder architecture to handle these critical issues. To this end, on the basis of the encoder-decoder architecture, we propose a brain-inspired spatially-varying adaptive pyramid context aggregation network. Concretely, with the proposed spatially-varying adaptive pyramid context aggregation module, every pixel location of the feature map is reweighted with the learned weight factor guided by the aggregated global contextual cues. Feature vectors of any two pixel locations are explicitly interacted by the form of matrix multiplication between the reshaped two-dimensional feature maps, leading to the spatially-varying feature weight factors. The generated weight factors are then utilized as the dilated depth-wise convolution kernels with different dilation factors to aggregate the contextual cues in receptive fields with multiple scales. In this way, the contextual cues are integrated into the feature maps with predictable and spatially-varying depth-wise convolutions. In addition, we employ a structured triplet ranking loss, whose aim is to supervise the network to enlarge the distance of feature embedding between non-matching keypoints and narrow the distance of feature embedding between the matching keypoints, leading to compactness between matching keypoints and dispersion between non-matching keypoints.

In order to verify the effectiveness of the proposed method, proper dataset and evaluation metric should be elaborately designed. However, current FIRE dataset (Hernandez-Matas et al., 2017) only labels a small part of the keypoints. Meanwhile, some keypoints of FIRE dataset are not located at branching or crossing points. So this dataset can't be used for training our proposed model. To this end, we collect 200 retinal images of 50 patients taken with fundus camera by RetCam3 and Canon. Concretely, 100 neonatal fundus images of 27 patients with low imaging quality are taken from RetCam3. Another 100 high-quality retinal images of 23 patients taken from Canon are also included. Meanwhile, different imaging angles and diverse overlapping areas between the image pair are also considered during the construction of dataset. In order to quantitatively evaluate the proposed method, following previous methods (Hernandez-Matas et al., 2017), we choose the Area Under Curve (AUC) value as the registration score. Experimental results demonstrate that our proposed method achieves significant performance improvement over the vanilla encoder-decoder network. Our method achieves the best registration performance among the deep learning based methods. Meanwhile, our proposed method also surpasses most of the traditional registration methods with significantly faster execution speed by an order of magnitude.

Our contributions are summarized into three parts:

- We propose to achieve **reliable** and **stable** keypoint detection and registration results for fundus image. Considering that the vessel crossing and branching points can reliably and stably characterize the key components of fundus image, we propose to learn to detect and match all the crossing and branching points of the input image pair with a single deep neural network.
- In order to cope with the large intra-class variability and small inter-class difference of retinal image, we propose a brain-inspired spatially-varying adaptive pyramid context aggregation based on the widely used encoder-decoder architecture. In this way, long-range contextual cues are incorporated into the feature maps with predictable and input-variant convolutions. Moreover, a structured triplet ranking loss is employed to enforce the network to produce similar feature embedding for matching keypoints in the input image pair, and dissimilar feature embedding for non-matching keypoints.
- Since there is no proper fundus image registration dataset for method evaluation, we construct a large-scale dataset which covers diverse application scenarios. Quantitative and qualitative results show that our proposed method is able to reliably and stably locate and match keypoints.

We organize our paper as follows. Section 2 reviews related work. Section 3 shows the detail of our method. Section 4 demonstrates experimental results. Finally, Section 5 presents our conclusion.

## 2. Related work

### 2.1. Deep learning based image registration

Since the learning based image registration is mainly considered in this paper, we provide a brief review of related works on deep learning based image registration in this part. In recent years, several methods (Cao et al., 2017; Krebs et al., 2017) have proposed to employ the DNN to directly learn the warp field between the test image and reference image. Ground truth warp fields are required in the above methods (Rohé et al., 2017; Sokooti et al., 2017; Yang et al., 2017) to supervise the learning of DNN. In order to obtain the ground truth warp field, several methods propose to simulate the deformation operation and generate deformed images. Some other methods employ the classical registration method, which rely on hand-designed feature. However, the above methods are difficult to obtain ground truth warp field as the ground reality, which severely limit the application in real scenario. Recently, several unsupervised learning based image registration methods (Li and Fan, 2017; Vos et al., 2017; Zou et al., 2020) are also proposed. However, these methods fail to cope with complex image registration application, such as large transformations (Vos et al., 2017).

Compared to images collected in our daily life, the retinal image registration is a much more challenging problem. First, there are large differences in illumination, color, contrast and imaging angles of the input image pair in diverse scenarios. The overlapping areas between the test image and the reference image may be also diverse. Furthermore, significant changes in retinal structure may be caused by the progression of retinopathy. As a result, current deep learning based image registration methods fail to achieve reliable and stable registration results, which are not applicable for the challenging fundus image task.

### 2.2. Deep metric learning

Deep metric learning aims to learn the distance metric to compare and measure similarity between pairs of examples, which is important for various tasks, such as image retrieval (Sohn, 2016; Movshovitz-Attias et al., 2017), clustering (Hershey et al., 2016). One of the main task of deep metric learning is to design proper loss function. Contrastive loss (Chopra et al., 2005; Hadsell et al., 2006) aims to encode the pair-wise relations between the anchor example and one similar(positive) or dissimilar(negative) example, which is first proposed to learn the feature embedding for image search task. Triplet loss (Wang et al., 2014; Schroff et al., 2015; Cui et al., 2016) is used to learn feature embedding for face recognition task. A triplet is composed of the anchor example, a positive example and a negative example. The triplet loss is to learn a distance metric by which the anchor point is closer to the similar point than the dissimilar one by a margin. Recently, richer structural relations among multiple examples are considered by ranking-motivated methods (Schroff et al., 2015; Oh Song et al., 2016; Sohn, 2016; Law et al., 2017; Movshovitz-Attias et al., 2017). Some other methods propose to design clustering-motivated structured losses (Hershey et al., 2016; Oh Song et al., 2017). However, since clustering-motivated losses are more difficult to optimize, the ranking-motivated loss function is mainly considered in this paper.

## 3. Method details

**Figure 1:**
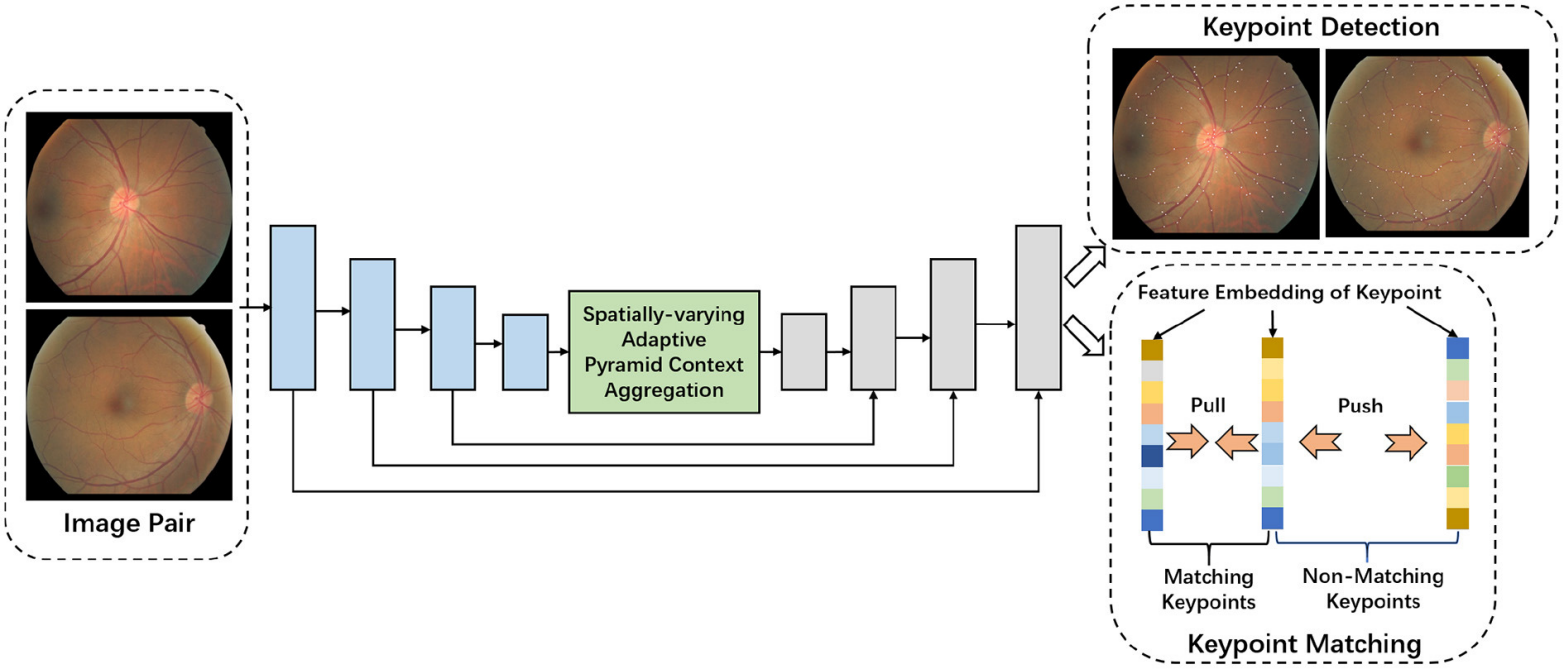
Overview of the proposed network for simultaneous keypoints detection and keypoints matching.

This section presents details of our method for reliable and stable fundus image registration. We show the overview of the proposed model in Figure 1. We start by introducing the encoder-decoder network, which is the baseline of our model. Then we introduce the proposed network architecture and employed loss function.

### 3.1. Encoder-decoder network architecture

For the fundus image registration method based on deep neural network (DNN), in order to achieve accurate pixel-level image registration results, robust global semantic information and rich local spatial details are required. Current DNN stacks successive convolutional and pooling layers to obtain roust feature representations. However, due to the multiple pooling operations, the feature spatial resolution is largely reduced. As a result, local spatial details are severely lost for the features in deeper-level layers. On the contrary, due to fewer pooling layers, spatial resolution of features in lower-level layers are larger. In this way, the features in lower-level layers encode rich local spatial details. However, the lack of semantic and discriminative cues make the lower-level features fail to effectively model long-range information. Since both the local spatial details and global semantic cues are essential for accurate image registration performance, a balanced fusion of the lower-level features and the deeper-level features is required.

As shown in Figure 1, current widely used encoder-decoder architecture employs the encoder sub-network to extract the multi-scale features by multiple stacked convolutional and pooling operations. The later decoder sub-network then combines the extracted multi-level features by multiple feature fusion operations. Concretely, with the input image pair, the successive convolutional and pooling layers of encoder sub-network extract multi-scale features, similar to ResNet (He et al., 2016) or VGGNet (Simonyan and Zisserman, 2014). The decoder sub-network consists of multiple feature fusion operations, which are employed to fuse the multi-scale features generated by the encoder sub-network progressively. For every fusion operation, $\hat{F_{i}}$, the feature in current layer *i*, is first upsampled to match the resolution of the feature map *F*_*i*−1_ from the lower neighbor layer *i*−1. The feature concatenation along the channel dimension is applied, which is followed by another convolution for further feature abstraction. This operation can be formulated as:


(1) $$\begin{array}{l} F_{{\;}_{i-1}}^{\text{\textasciicircum{}}}=Conv(Concat(Up(({\hat{F}}_{i})),F_{i-1})). \end{array}$$

The above fusion operation is iterated until the lowest layer, where the generated feature *F*_1_ has the same spatial resolution as the input image, which is used to produce the final prediction.

### 3.2. Spatially-varying context aggregation module

Due to the large intra-class variability and small inter-class difference of fundus image, the non-matching keypoints are prone to be misclassified. As a result, contextual cues should be incorporated into the vanilla encoder-decoder architecture to handle this critical issue (Liu et al., 2020). To this end, with the deepest feature map generated by the encoder, a novel context aggregation module is applied to incorporate the contextual cues in a spatially-varying manner. The details are illustrated below.

In order to model the long-range contextual cues, previous methods are mainly designed to generate global-consistent feature re-weighting coefficient. For example, SE-Net (Jie et al., 2019) is proposed to produce channel-wise feature re-weighting factor of global distribution by a squeeze-and-excitation mechanism. Differently, we propose to aggregate the global contextual cues by generating spatially-varying feature re-weighting factors. In this way, the long-range relations are more effectively mined in a spatially-varying manner.

**Figure 2:**
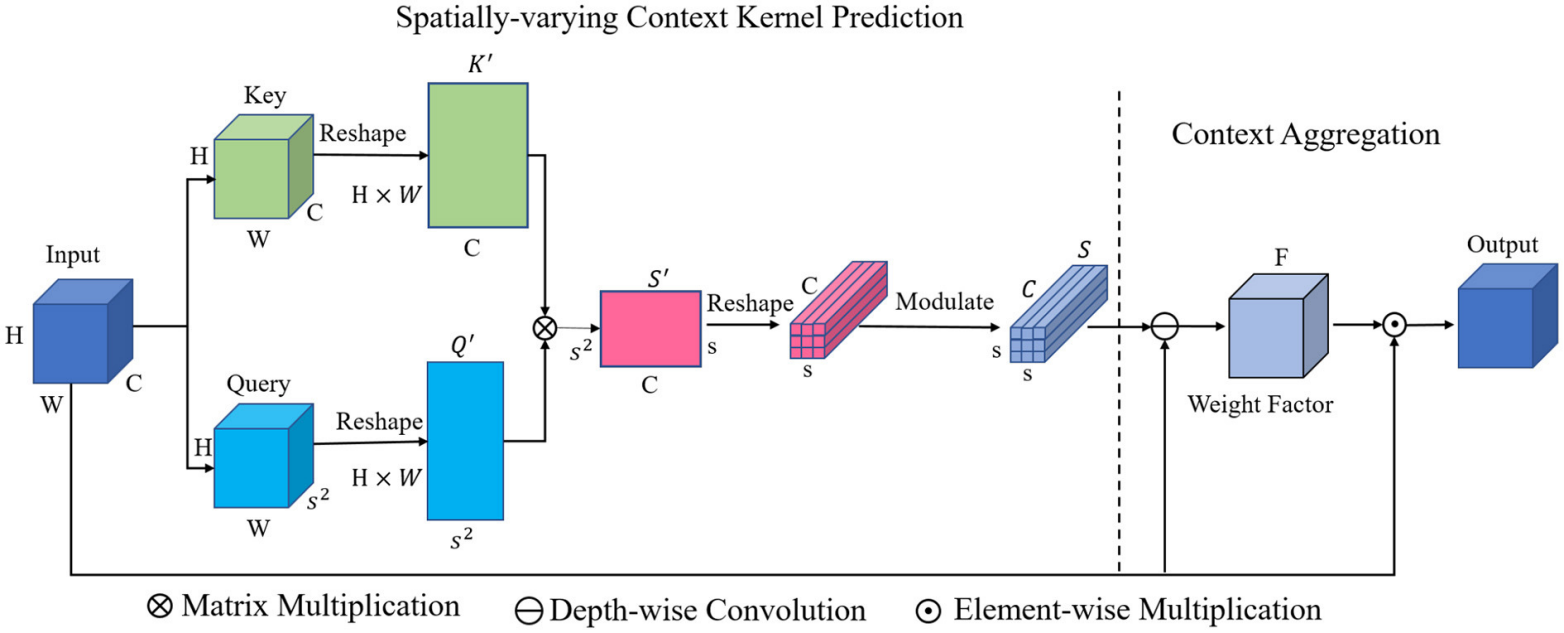
Details of the proposed spatially-varying context aggregation module, which first predicts the spatially-varying context kernel and then aggregates the context with the predicted re-weight kernels.

Figure 2 shows the overall architecture of the proposed Spatially-varying Context Aggregation (SCA) module. First, we explicitly model the long-range relations between any two pixel locations by matrix multiplication, generating spatially-varying context kernel prediction. Then, the predicted context kernels are applied on the original feature map, leading to aggregated context enhanced feature. Following are the detailed processing pipeline.

With the feature map *X* ∈ *R*^*H*×*W*×*C*^ generated by the last feature block of the encoder, we first transform it into two forms with two independent convolutional operations: the *key* and *query*. The *H*, *W* and *C* refer to the hight, width and channel number, respectively. The *key* feature map *K* ∈ *R*^*H*×*W*×*C*^ and the *query* feature map *Q* ∈ *R*^*H*×*W*×*s*^2^^ are then used to aggregate the contextual cues. Here, *s* is the kernel size of the learned context kernel.

In order to effectively model the global contextual cues between pixels, the relation within any pixel locations should be explicitly interacted. To this end, the *key* feature map *K* ∈ *R*^*H*×*W*×*C*^ and the *query* feature map *Q* ∈ *R*^*H*×*W*×*s*^2^^ are first reshaped into 2D form, *K* ∈ *Q*^*H*×*W*×*C*^ and *Q*′ ∈ *R*^(*H*×*W*) × *s*^2^^, respectively. In this way, our aim is to make each column of *K* effectively encodes the channel-wise characteristics of original feature map *X* along the channel dimension *C*. The length of each of the *C*−dimensional feature vector is *H*×*W*. Meanwhile, each column of *Q*′ models one of the *s*^2^-dimensional feature vectors with the length of *H*×*W*.

Afterwards, in order to explicitly model the interactions between each column of *K* ∈ *Q*^*H*×*W*×*C*^ and *Q*′ ∈ *R*^(*H*×*W*) × *s*^2^^ for all the (*H*×*W*) pixel locations, we employ following operations:


(2) $$\begin{array}{l} S^{{\;}^{\prime }}\left(i,j\right)=\overset{H\times W}{\underset{q=1}{\sum }}Q^{{\;}^{\prime }}\left(q,i\right)\times K^{{\;}^{\prime }}\left(q,j\right), \end{array}$$

Where *i* = 1, 2, ....., *s*^2^, *j* = 1, 2, ...., *C*. Since the number of *query* vectors is *s*^2^, *s*^2^ feature vectors encoded the interactions between all the pixel locations can be thus obtained. The length of each of the feature vector is *C*. We can also rewrite the above operation of dot product form as a form of matrix multiplication:


(3) $$\begin{array}{l} S^{{\;}^{\prime }}={Q^{{\;}^{\prime }}}^{T}\times K^{{\;}^{\prime }}, \end{array}$$

Where *Q*′^*T*^ refers to the transpose of matrix *Q*′, *S*′ ∈ *R*^*s*^2^×*C*^ is the union of all the obtained cues about spatial location relation.

Then, the generated two-dimensional *S*′ ∈ *R*^*s*^2^×*C*^ is reshaped into 3D form *S* ∈ *R*^*s*×*s*×*C*^. We then employ a batch normalization operation to modulate *S*, generating the predicted spatially-varying context kernel. The generated kernel effectively encodes the relation cues between pixels of all spatial locations, which can be used to produce spatially-varying weight factor *F* ∈ *R*^*H*×*W*×*C*^ for all *H*×*W* spatial locations.

**Figure 3:**
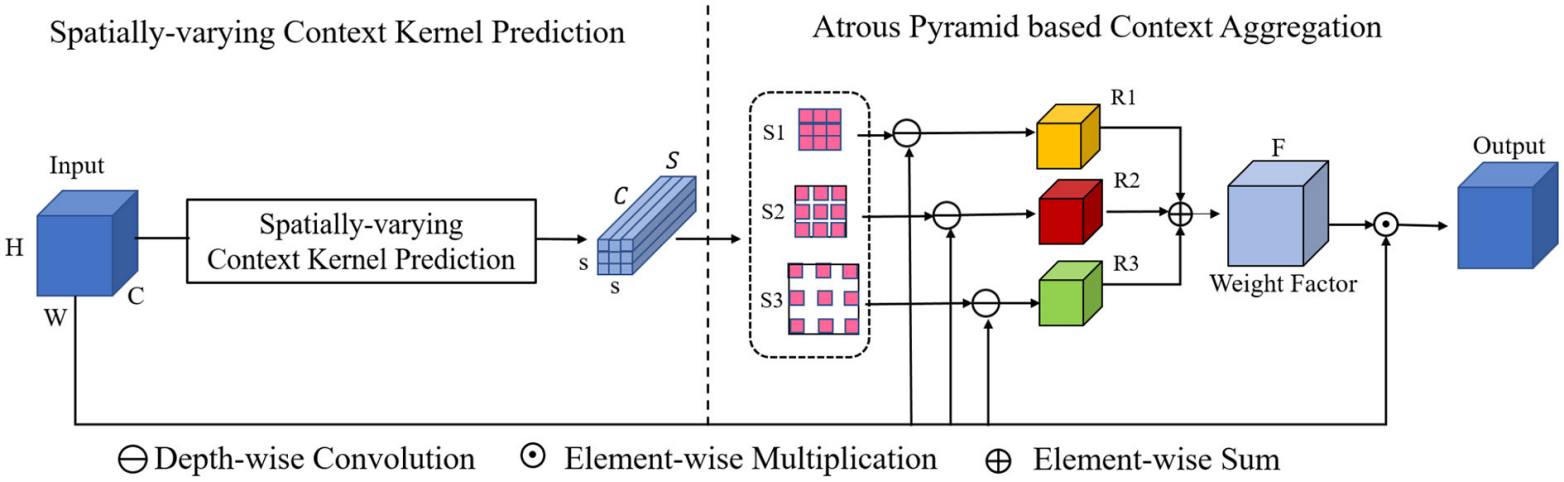
Details of the proposed spatially-varying adaptive pyramid context aggregation module. The adaptive pyramid mechanism aggregates the contextual cues with multi-scale field-of-views via convolution pyramid with multiple atrous rates.

In order to fully exploit the information encoded in the spatially-varying context kernel, the depth-wise convolution is applied on the original feature map *X* with the context kernel *S* as the depth-wise convolution kernel. In this way, each channel of *S* is able to modulate one specific channel of *X* in an independent manner. The spatially-varying context guided modulation can thus be implemented. Concretely, as shown in Figure 3, we first split the context kernel *S* ∈ *R*^*s*×*s*×*C*^ into *C* two-dimensional kernels along the channel dimension. Each of the 2D *C* kernels has a spatial dimension of *s*×*s*. These *C* kernels are then applied on each channel of the original feature map *X* ∈ *R*^*H*×*W*×*C*^ in an independent manner, generating intermediate feature. A 1 × 1 × 1 convolution is then used to transform the generated intermediate feature map for further feature abstraction. The obtained feature is then processed with one Sigmoid activation function, which produces the spatially-varying weight factor *F* ∈ *R*^*H*×*W*×*C*^. Finally, an element-wise multiplication between *M* and *X* is performed to achieve the output feature map, which is then passed through the decoder part for multi-scale feature fusion.

### 3.3. Spatially-varying adaptive pyramid context aggregation module

#### 3.3.1. Dilated convolution

Standard convolution is characterized by its property of local receptive field. However, large receptive field is essential for enhancing deep neural network's discriminative feature learning ability. Hence, pooling layer is used after several convolutional layers to enlarge the receptive field. However, the adoption of pooling layer leads to the loss of spatial details and lower-resolution feature map, which is unfavorable for accurate pixel-level keypoint location and matching. Dilated convolution is able to effectively alleviate this challenging issue by sparsifying the standard convolution separated by zero with specific interval (dilation rate), which allows us to enlarge the receptive field without loss of spatial resolution of the feature map.

#### 3.3.2. Depth-wise dilated convolution

Depth-wise separable convolution transforms the standard convolution into a depth-wise convolution followed by a point-wise convolution. In this way, the computation complexity is thus drastically reduced. Concretely, the depth-wise convolution is applied on each channel of the feature map independently. The point-wise convolution is then used to fuse the output from the depth-wise convolution.

On the basis of the context aggregation module above, a dilation pyramid based context aggregation module is incorporated for further context aggregation of multi-scale field-of-view, as shown in Figure 3. Concretely, with the predicted spatially-varying context kernel *S*, we employ three parallel dilated convolutions with different dilation rates to model contextual cues in a context-adaptive manner. The three different dilation rates are set as 1, 3, 5 in our paper. In this way, a dilation pyramid context aggregation block is obtained.

With these operations, three context kernels (*S*1, *S*2, and *S*3) with different context aggregation fields are obtained. The three context kernels are then applied over the original feature map *X*, leading to three different weight factors *R*_1_, *R*_2_, and *R*_3_. The three generated weight factors are then fused by element-wise sum:


(4) $$\begin{array}{l} R=R_{1}+R_{2}+R_{3}. \end{array}$$

With the final fused weight kernel *R*, similar to the above SCA module, an element-wise multiplication is operated between *R* and *X* to ensure each channel of *R* can independently modulate the corresponding channel of *X*.

### 3.4. Loss function

In order to supervise the above network to effectively locate and match the keypoints, specifically designed loss functions are utilized.

#### 3.4.1. Keypoint location loss

We convert the keypoint location task into a pixel-level binary classification problem. In order to accurately locate the keypoints, the widely used cross-entropy loss is first utilized to supervise the learning of the transformed feature map of the last feature block of the above adaptive pyramid context aggregation network:


(5) $$\begin{array}{l} CE\left(y_{i},p_{i}\right)=-\left[y_{i}log\left(p_{i}\right)+\left(1-y_{i}\right)log\left(1-p_{i}\right)\right], \end{array}$$

Where *y*_*i*_ means the label of pixel i (1 and 0 means the keypoint and background, respectively), *p*_*i*_ refers to the predicted probability of pixel i to be the keypoint.

We also use the Dice loss for more accurate keypoint location:


(6) $$\begin{array}{l} Dice\left(X,Y\right)=1-\frac{2|P\cap Y|}{\left|P|+|Y\right|}, \end{array}$$

Where P means the pixel set of the predicted keypoints, Y means the pixel set of ground truth keypoints. |*P*∩*Y*| refers to the sum of the element-wise production between *P* and *Y*. |*P*|+|*Y*|, |*P*| refers to the sum of all the elements of *P*, |*Y*| refers to the sum of all the elements of *Y*.

#### 3.4.2. Keypoint matching loss

In order to supervise the network to enhance the discriminative power of learned feature embedding of keypoints, proper keypoint matching loss should be designed. The ideal keypoint matching loss should reduce the gap between matching keypoints and enlarge the gap between non-matching keypoints.

To this end, with the feature map in the last feature block of decoder before generating keypoint detection prediction, we transform this feature map into three-dimensional feature embedding. Thus, every fundus image keypoint has its corresponding one-dimensional feature embedding. Following Huang et al. (2016) and Opitz et al. (2017), we set the feature embedding dimension as 512. In this way, our task is to enlarge the distance of feature embedding between non-matching keypoints and narrow the distance of feature embedding between the matching keypoints, leading to compactness between matching keypoints and dispersion between non-matching keypoints. Metric learning mechanism is employed to tackle the above problem in this paper. Concretely, we use the ranking loss to compute the relative distance between the one dimensional feature embedding of every two keypoints in the input image pair.

##### 3.4.2.1. Pair-wise ranking loss

This widely used loss is also called contrastive loss. Positive and negative pairs of the one-dimensional feature embedding of keypoints in input image pair are both required for computing the pair-wise ranking loss. One positive pair consists of an anchor keypoint *k*_*a*_ and the matching keypoint *k*_*p*_. One negative pair consists of an anchor keypoint and a non-matching keypoint *k*_*n*_. The one-dimensional feature embedding of the anchor keypoint *k*_*a*_, the matching keypoint *k*_*p*_ and the non-matching keypoint *k*_*n*_ are *f*_*a*_, *f*_*p*_, and *f*_*n*_, respectively. For positive pairs, the aim of the pair-wise ranking loss is to guide the network to learn proper feature embedding with a small distance. On the contrary, for negative pairs, the pair-wise ranking loss aims to supervise the network to learn feature embedding with a large distance. We choose the Euclidian distance as the distance computing function to measure the similarity between the feature embedding. The above operations can be formulated as:


(7) $$\begin{array}{l} L\left(f_{a},f_{p},f_{n}\right)=\{\begin{array}{rr} d\left(f_{a},f_{p}\right), & if\;PostivePair,\\ max\left(0,m-d\left(f_{a},f_{n}\right)\right), & if\;NegativePair. \end{array} \end{array}$$

As shown in the Equation 7, for one positive pair, if the distance between *f*_*a*_ and *f*_*p*_ are larger than 0, the loss value will also be positive. Hence, the network is guided to reduce the distance to be 0. In this way, this pair-wise ranking loss guides the network to produce similar feature embedding for matching keypoints. On the other hand, for negative pair, when the distance between the feature embedding of the anchor keypoint and negative (non-matching) keypoint is larger than a specific margin threshold, the loss will be 0. When the distance is reduced below the margin value, the loss value will be positive. When the distance between *f*_*a*_ and *f*_*p*_, the loss value is the largest value *m*. In this way, the pair-wise ranking loss supervises the network to produce dissimilar feature embedding for non-matching keypoints. When the distance for a negative pair is distant enough (larger than the default threshold), the network will focus on the learning of feature embedding for more difficult pairs.

##### 3.4.2.2. Triplet ranking loss

Instead of using only one pair of keypoints for every computation of pair-wise ranking loss, the triplet ranking loss considers the relations of a triplet, which consists of an anchor keypoint *k*_*a*_, a positive keypoint *k*_*p*_ and a negative keypoint *k*_*n*_. The aim of the triplet ranking loss is to guide the network to produce separable feature embedding: the distance between the feature embedding of the anchor keypoint and negative keypoint *d*(*f*_*a*_, *f*_*n*_) is larger than the distance between the feature embedding of anchor keypoint and the positive keypoint *d*(*f*_*a*_, *r*_*p*_) by a specific margin *m*). The above operations can be rewritten as:


(8) $$\begin{array}{l} L\left(f_{a},f_{p},f_{n}\right)=max\left(0,m+d\left(f_{a},f_{p}\right)-d\left(f_{a},f_{n}\right)\right). \end{array}$$

We note that the difference between the pair-wise ranking loss and triplet ranking loss is that pair-wise ranking loss only considers pair of keypoints for one loss computation, however, a triplet of anchor keypoint, positive keypoint and negative keypoint is considered for the triplet ranking loss.

##### 3.4.2.3. Structured triplet ranking loss

Triplet loss (Weinberger and Saul, 2009; Schroff et al., 2015) is proposed to pull the learned feature embedding of anchor keypoint closer to the positive keypoint than to the negative keypoint by a fixed margin. However, the triplet loss only considers one triplet for every loss computation, neglecting the relations among multiple keypoints. To this end, inspired from Oh Song et al. (2016); Wang X. et al. (2019), we propose to employ the structured triplet ranking loss to supervise the feature embedding learning of our network, which explores the structured relationship among multiple keypoints.

Concretely, the structured triplet ranking loss encourages the interaction between more negative keypoints. On the basis of triplet loss, the employed structured triplet ranking loss aims to supervise the learned feature embedding between the anchor keypoint and one positive keypoint is as similar as possible. Moreover, the feature embedding between the anchor keypoint and all negative keypoints as dissimilar as possible. Formally, the structured triplet ranking loss aims to pull the anchor keypoint closer to one positive keypoint than all negative keypoints than a margin *m*.


(9) $$\begin{array}{l} L=\frac{1}{2|P|}\sum_{(i,j)\in P}\left[d(f_{i},f_{j}\right)+log(\sum_{(i,p)\in N}exp(m-d(f_{i},f_{p}))\\ \;+\sum_{(j,l)\in N}exp(m-d(f_{j},f_{l})){]}_{+}, \end{array}$$

**Figure 4:**
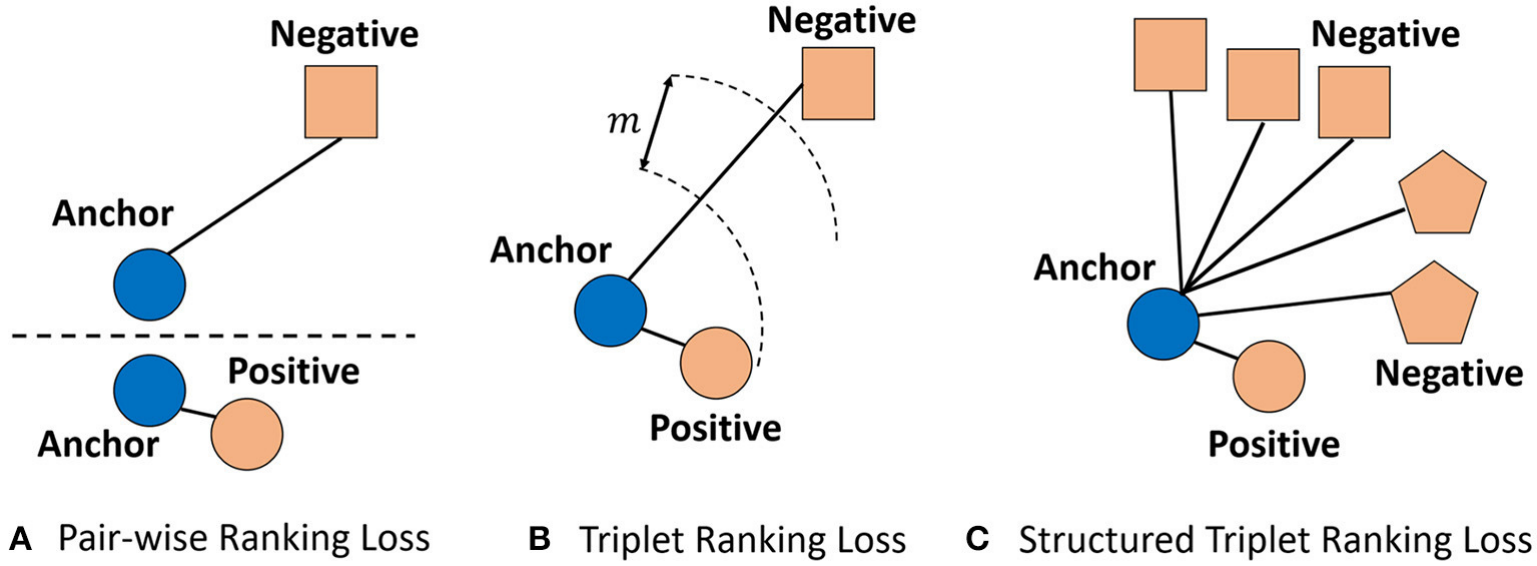
Illustration of the **(A)** Pair-wise Ranking loss, **(B)** Triplet Ranking loss, and **(C)** Structured Triplet Ranking loss. Different shapes represent different classes. The blue circle is an anchor. For Pair-wise Ranking loss, the anchor and one positive example or one negative example are considered for every loss computation. For Triplet Ranking loss, the anchor is compared with only one negative example and one positive example. For the Structured Triplet Ranking loss, the anchor is compared with all negative examples.

Where **P** and **N** are the set of positive pairs and negative pairs respectively, *f*_*i*_, *f*_*p*_, *f*_*j*_, and *f*_*l*_ refer to the feature embedding of pixel *i*, pixel *p*, pixel *j*, and pixel *l*, respectively. [·]_+_ is the hinge function. Illustration of the Pair-wise Ranking loss, Triplet Ranking loss, and Structured Triplet Ranking loss are shown in Figure 4.

### 3.5. Implementation details

The hyperparameters of batch-size, weight decay are set to 1, 1*e*−3 respectively. The monmentum is set as 0.9. We use pytorch (Paszke et al., 2017) as the basic implement architecture. The widely used stochastic gradient descent strategy is used for training the proposed model.

## 4. Experiments

In this section, we present extensive experiments to validate the proposed model for fundus image registration. First, we show our evaluation dataset and metric. Then we present a detailed analysis of our model on the constructed large-scale dataset.

### 4.1. Datasets and metrics

#### 4.1.1. Dataset

Current widely used funds image registration dataset, FIRE, consists of 134 image pairs from 39 patients, which are acquired with Nidek AFC-210 fundus camera. The keypoints of images in FIRE dataset are randomly labeled in a sparse manner. There is not a guarantee that all the vessel branching and crossing points are labeled as keypoints. In this case, these sparse ground-truth keypoint labelings fail to train our proposed model. As a result, a large-scale fundus image registration dataset, which labels all the keypoints in a reliable and stable manner, is required for further research.

**Figure 5:**
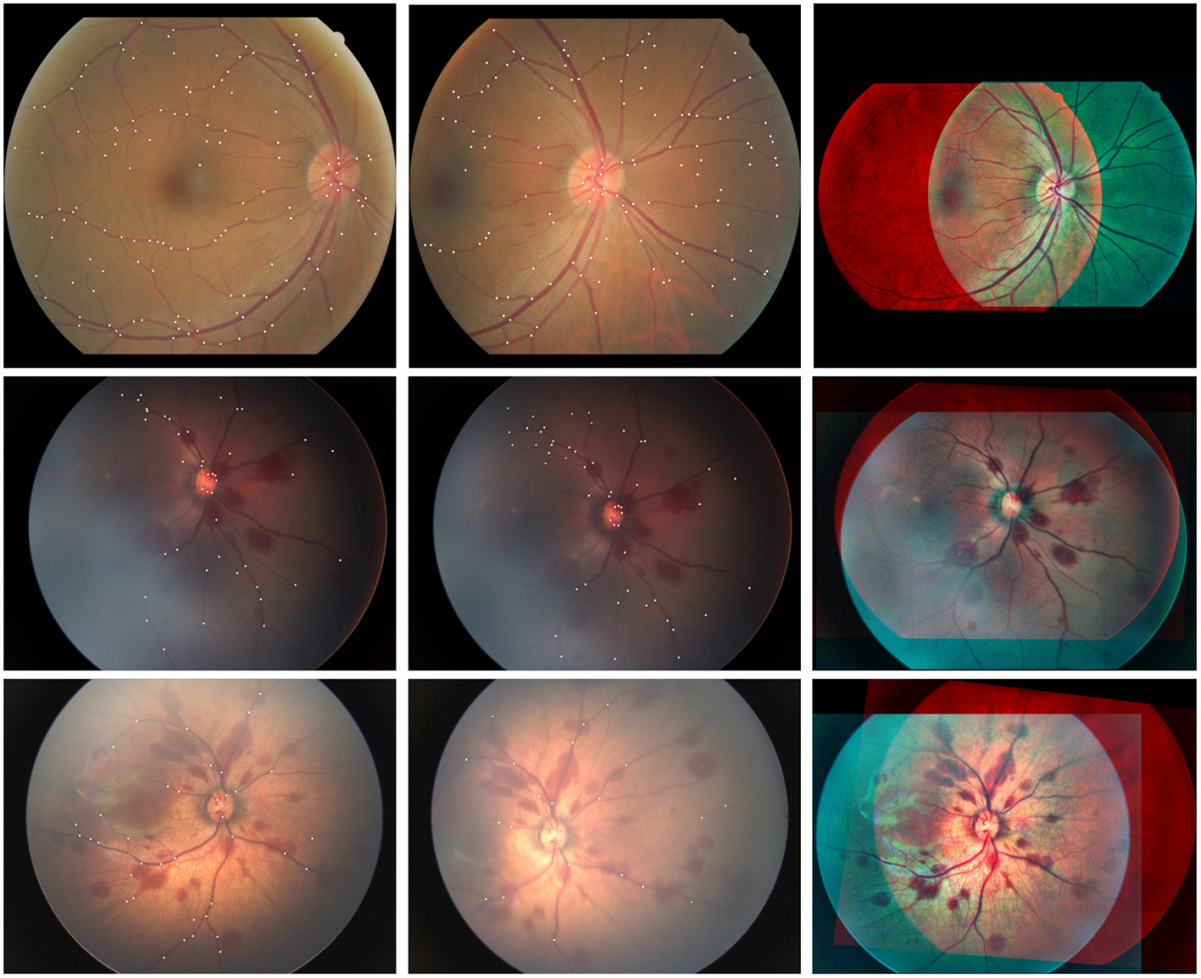
Example of the fundus images from diverse applications, including adult and neonatus patients acquired under good or bad imaging conditions. Moreover, different imaging angles and overlapping areas between the image pairs are also considered.

To this end, we collect 200 pairs of fundus images under various imaging conditions (illumination, angle etc.) taken from different fundus cameras, such as Canon and RetCam3, as shown in Table 1. The constructed dataset is termed as AN-200 dataset. Concretely, 100 high-quality retinal images of 27 adult patients are acquired from Canon. Moreover, the neonatal fundus images are often with low image quality, due to the uncooperative image acquiring process. We collect 100 neonatal fundus images taken from 23 patients with RetCam3 to support various neonatal applications. In addition, different imaging angles and lighting conditions are considered during the construction of the dataset. Example of the fundus images are shown in Figure 5. For every image pair, all the branching and crossing points are labeled as keypoints. All the matched keypoints are then labeled as ground truth matching keypoints. In this way, a reliable and stable fundus image registration dataset is constructed.

**Table 1:** Details of our constructed AN-200 dataset.

	**Camera**	**Number of image pairs**	**Number of patients**
Adult	Canon	100	27
Neonatus	RetCam3	100	23

We collect and label 200 fundus image pairs of adult and neonatus, which are taken from Canon and RetCam3.

#### 4.1.2. Evaluation metric

First, we choose the widely used FIRE dataset to quantitatively evaluate the proposed method and compare with state of the art methods. Since FIRE dataset only labels part of the crossing and branching points, our model cannot be trained on this dataset. Following Rivas-Villar et al. (2022), we train the models on the training set of our constructed dataset. The trained models are then evaluated on FIRE dataset with the registration score proposed by Hernandez-Matas et al. (2017), which calculate the success ratio between the fixed and moving image pairs after the transformation of the moving image with the learned transformation parameters.

Concretely, pixels of moving image are first transformed into the coordinate space of fixed image. We then calculate the averaged distance between the transformed pixels and the ground-truth points of fixed image as the registration error of this image pair. If the registration error is below a threshold, the registration of this image pair is successful. With larger threshold, more image pairs are deemed successful registrations. By varying the threshold from 0 to larger value, the percentage of successful registration pairs enlarges gradually. In this way, we can plot the registration curve, where the X axis corresponds to the setting threshold, the Y axis refers to the percentage of successfully registered images. With the plotting curve, the Area Under Curve (AUC) can be calculated as the final registration score. The original FIRE dataset (Hernandez-Matas et al., 2017) is divided into three sub-datasets based on the overlapping and anatomical similarity between an image pair. The sub-dataset *S* consists of 71 image pairs with more than 75% overlapping and no anatomical differences. The sub-dataset *P* contains 49 image pairs with less than 75% overlapping. Finally, the sub-dataset *A* is composed of 14 image pairs with anatomical differences. Similar to Rivas-Villar et al. (2022), we calculate the AUC score on the *S*, *P*, and *A* sub-datasets and the whole FIRE dataset.

In addition, we also calculate the AUC value as the registration score on our constructed AN-200 dataset with the same computing manner. Concretely, 60%, 20% and 20% of the original dataset are randomly divided into the training, validation and test set, respectively. The final registraction score is reported on the test set.

### 4.2. Ablation study on the network architecture

Based on the constructed dataset, in order to obtain better understanding of the proposed network, we evaluate following methods with different network settings. The experimental results are summarized in Table 2:

- Baseline: We first choose the vanilla encoder-decoder architecture (U-Net) as the backbone network to simultaneously learn the detection of keypoint and the generation of feature embedding, under the supervision of the above cross-entropy loss, Dice loss and the proposed structured triplet ranking loss. As shown in Table 2, the Baseline achieves an AUC of 70.5 and 68.1% on AN-200 and FIRE datasets, respectively.
- Spatially-varying context aggregation network (SCA-Net): Then we enhance the simple U-Net with the proposed spatially-varying context aggregation module. Concretely, over the last stage of the encoder sub-network of U-Net, the generated feature map of encoder sub-network is enhanced with the SCA module. The global contextual cues are thus incorporated. The loss functions are kept the same with the Baseline. The AUC on AC-200 of SCA-Net is 72.2%, and the AUC on FIRE is enlarged to 69.5%. The performance improvement is 1.7 and 1.4%, respectively.
- Spatially-varying adaptive pyramid context aggregation network (SAPCA-Net): Finally, we test our overall network, SAPCA-Net, by changing the SCA-module with the SAPCA module to incorporate context-adaptive cues. Compared to original U-Net, the SAPCA-Net largely improves the AUC of AD-200 by 2.4%, the AUC of FIRE by 3.0%. Concretely, the AUC of AD-200 is significantly enlarged from 70.5 to 72.9%, and the AUC of FIRE is improved from 68.1 to 71.1%. These results effectively show the effectiveness of the proposed SAPCA module.

**Table 2:** The evaluation results of methods with different network settings on AN-200 and FIRE datasets.

**Method**	**AN-200(%)**	**FIRE(%)**
U-Net	70.5	68.1
SCA-Net	72.2	69.5
SAPCA-Net	**72.9**	**71.1**

The bold values mean the best performance.

**Figure 6:**
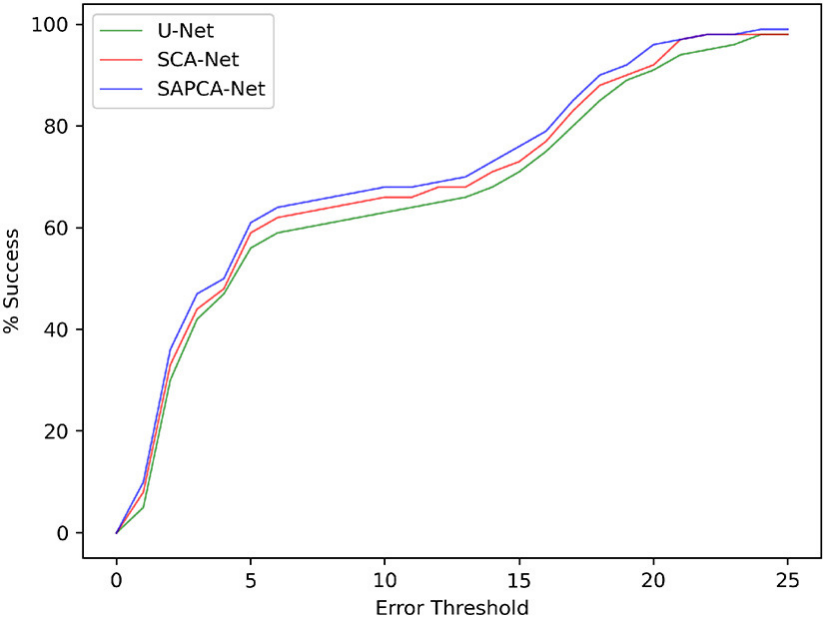
The registration success with different error thresholds for the model with different network settings on FIRE dataset.

**Figure 7:**
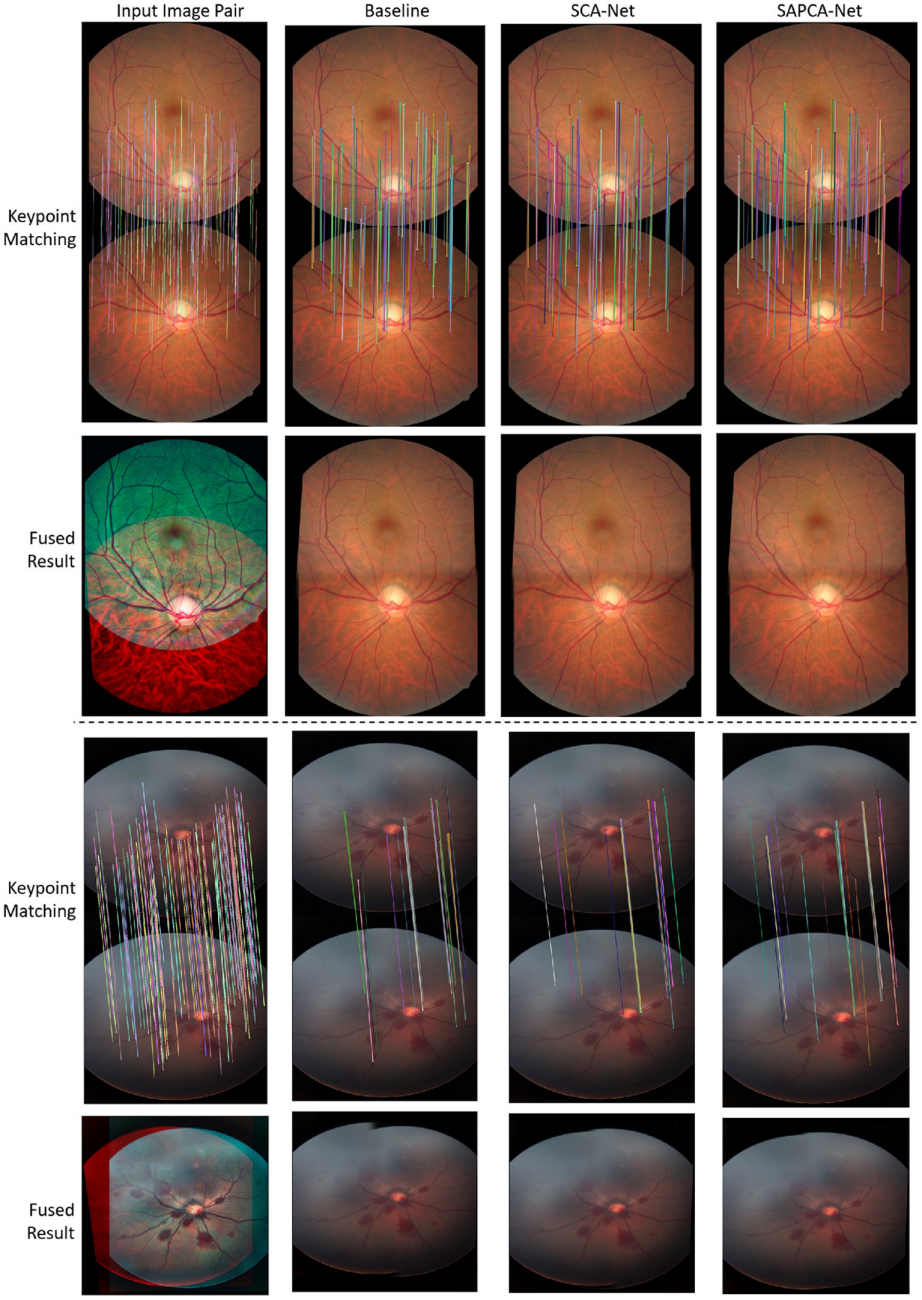
Example of the keypoint detection and matching results of normal adult and neonatal fundus images. We also show the fused image with the matching keypoints.

As shown in Figure 6, we plot the curve of the successful registration ratio as the change of different error thresholds. In addition to the above quantitative comparisons, we also show the visualized results of our method. Figure 7 demonstrates the visualized keypoint detection and keypoint matching results from two typical scenarios. The last row also shows the final fused results with the matching keypoints. The first column of Figure 7 shows the ground truth keypoint detection and fused result. As shown in Figure 7, the baseline method is able to effectively locate and match keypoints. However, there exist a number of wrong keypoint matching results. The SCA-Net is able to remove some false positive predictions, leading to better keypoint matching result. Finally, the SAPCA-Net further removes more false positive keypoint matching predictions. Meanwhile, the number of true keypoint matching is also increased. As a result, the final fused result with the matching keypoints generated by the SAPCA-Net is visually better than other methods. These qualitative comparisons further demonstrate the effectiveness of the proposed network architecture.

### 4.3. Ablation study on the loss function

On the basis of the above best performing SAPCA-Net, we also conduct further ablation study for further understanding of the loss function. We evaluate the SAPCA-Net with following different loss functions, the results are summarized in Table 3:

- SAPCA-net-pairwise: We first replace the keypoint matching loss function of SAPCA-Net with the simple pairwise ranking loss. Pairwise ranking loss guides the SAPCA-Net to learn the pairwise relationship between the feature embedding of the anchor keypoint and one positive/negative keypoint. As shown in Table 3, the SAPCA-Net-Pairwise achieves the AUC of 71.4 and 69.7% on AN-200 and FIRE, respectively.
- SAPCA-net-triplet: Then we replace the keypoint matching loss function with the triplet ranking loss. The triplet loss helps the network to pull the anchor point closer to the similar keypoint than the dissimilar one by a margin. The AUC of SAPCA-Net-Triplet on AN-200 is 72.2%, and the AUC on FIRE is improved to 70.3%.
- SAPCA-net-structured-triplet: We further replace the keypoint matching loss function with structured triplet ranking loss. The structured triplet ranking loss supervise the network to learn the structured relationship among multiple keypoints. Compared to original pair-wise ranking loss, the AUC of AN-200 is enlarged from 71.4 to 72.9%, and the AUC of FIRE is improved from 69.7 to 71.1%. These results effectively show the effectiveness of the employed structured triplet ranking loss.

**Table 3:** Ablation study on the loss function.

**Method**	**AN-200(%)**	**FIRE(%)**
SAPCA-Net-Pairwise	71.4	69.7
SAPCA-Net-Triplet	72.2	70.3
SAPCA-Net-Structured-Triplet	**72.9**	**71.1**

With the structured triplet loss, SAPCA-Net-Structured-Triplet achieves the best result. The bold values mean the best performance.

**Figure 8:**
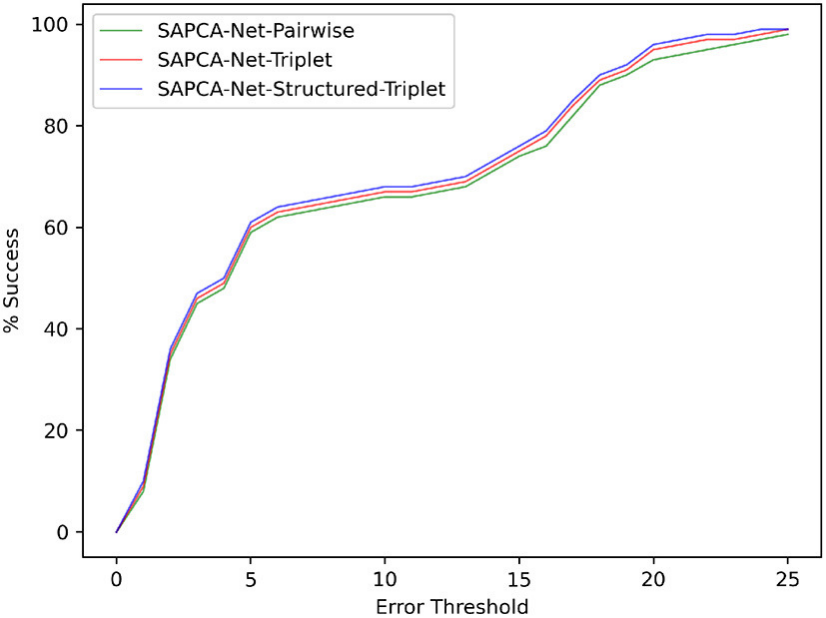
The curve of successful registration percentage under different error thresholds for the model with different loss functions on FIRE dataset.

Among the SAPCA-Net with the above three different loss functions, the SAPCA-Net-Structured-Triplet achieves significantly better results, which effectively demonstrates the superiority of the structured triplet ranking loss for the learning of matching keypoints. The change curve of registration success ratio under different error thresholds is shown in Figure 8.

### 4.4. Comparison to state-of-arts

In order to compare our proposed best-performing SAPCA-Net with state of the art methods, the widely used FIRE dataset is employed for evaluation. We first focus on the deep learning based methods. As shown in Table 4, compared to previous two-stage UNet + RANSAC (Rivas-Villar et al., 2022), our end-to-end registration method achieves consistently better results on the S, P, A sub-datasets and the whole FIRE dataset. Concretely, on the four dataset settings, our SAPCA-Net achieves the registration score of 93.9, 36.2, 71.9, and 71.1%, significantly outperforming UNet + RANSAC by 3.1, 6.9, 5.9, and 5.4%, respectively. Moreover, our model accomplishes the two steps of keypoint detection and matching with a single network. However, for previous UNet + RANSAC model, the keypoint detection is first accomplished by a U-Net, which is followed by traditional RANSAC (Fischler and Bolles, 1981) for the keypoint matching step. In this way, the execution time of our proposed SAPCA-Net is much shorter.

**Table 4:** Comparison to state-of-arts on FIRE dataset.

**Method**	**S**	**P**	**A**	**FIRE**	**Execution time**
SIFT + WGTM (Lowe, 2004)	83.7	54.4	40.7	68.5	–
GDB-ICP (Yang et al., 2007)	81.4	30.3	30.3	57.6	19
Harris-PIIFD (Yang et al., 2007)	90.0	9.0	44.3	55.3	13
SURF + WGTM (Bay et al., 2008)	83.5	6.1	6.9	47.2	–
ED-DB-ICP (Tsai et al., 2009)	60.4	44.1	49.7	55.3	44
RIR-BS (Chen et al., 2011)	77.2	0.49	12.4	44.0	-
ATS-RGN (Serradell et al., 2014)	36.9	0.0	14.7	21.1	-
EyeSLAM (Braun et al., 2018)	30.8	22.4	26.9	27.3	7
GFEMR (Wang J. et al., 2019)	81.2	60.7	47.4	70.2	10
RIFT + NTG (Zhou et al., 2022)	90.7	51.2	81.0	71.7	-
VOTUS (Motta et al., 2019)	93.4	67.2	68.1	81.2	106
REMPE (Hernandez-Matas et al., 2020)	95.8	54.2	66.0	77.3	198
U-Net + RANSAC (Rivas-Villar et al., 2022)	90.8	29.3	66.0	65.7	0.65
Our SAPCA-Net	93.9	36.2	71.9	71.1	0.32

Then, we compare our method with traditional registration methods. As shown in Table 4, our SAPCA-Net obtains the best registration score on the A sub-dataset, by achieving 71.1% AUC. This result is 3.8% better than previous best performing VOTUS. On the S sub-dataset, our method obtains the registration score of 93.9%, slightly better than VOTUS, while is 1.9% lower than the REMPE. On the whole FIRE dataset, our method outperforms most of the traditional methods. Although VOTUS and REMPE achieve better registration scores than our SAPCA-Net, the execution time of these two methods are two orders of magnitude slower than our method. Concretely, the execution time of our method is only 0.32s, which shows significant advantage compared to the VOTUS (106s) and REMPE (198s). This is a big advantage for applications in clinical scenarios.

## 5. Conclusion

Current deep learning based image registration methods directly learn to align the geometric transformation or the dense displacement vector field between the input image pair. These previous modeling paradigms fail to achieve keypoint detection and registration results in a reliable and stable way. To this end, in this paper, we aim to tackle this challenging issue. First, considering that the vessel crossing and branching points can reliably and stably characterize the key components for fundus image, a single network is employed to simultaneously learn to detect and match all the crossing and branching points of the input image pair in an end-to-end manner. Moreover, a spatially-varying adaptive pyramid context aggregation network is proposed to aggregate contextual cues in multi-scale field-of-view, which are much beneficial for accurate keypoint detection and matching. Furthermore, a structured triplet ranking loss is employed to guide the learning of similar feature embedding for matching keypoint and dissimilar feature embedding for non-matching keypoints. The proposed model is trained on a new constructed large-scale dataset with well-labeled ground-truths. Both quantitative and qualitative results show the effectiveness of the proposed method.

## Data availability statement

The raw data supporting the conclusions of this article will be made available by the authors, without undue reservation.

## Author contributions

JX and YC pointed out the problem of current methods and provided new solution. YC and PS designed the dataset constructing scheme. JX, YC, PS, LD, RS, and ZY collected and labeled the dataset. YC, PS, and DZ cleaned the dataset. JX, KY, LD, DZ, RS, and ZY performed the experiments. JX, YC, and PS evaluated the experimental results. KY wrote the first draft of the manuscript. All the authors revised the manuscript, contributed to the article, and approved the submitted version. All the authors approve the final version to be published and agree to be accountable for all aspects of the work in ensuring that questions related to the accuracy or integrity of any part of the work are appropriately investigated and resolved.

## References

[B1] Bay H. Ess A. Tuytelaars T. Van Gool L. (2008). Speeded-up robust features (surf). Comput. Vis. Image Understand. 110, 346–359. 10.1016/j.cviu.2007.09.014

[B2] Braun D. Yang S. Martel J. N. Riviere C. N. Becker B. C. (2018). Eyeslam: Real-time simultaneous localization and mapping of retinal vessels during intraocular microsurgery. Int. J. Med. Rob. Comput. Assist. Surg. 14, e1848. 10.1002/rcs.1848 28719002 PMC5762412

[B3] Cao X. Yang J. Zhang J. Nie D. Kim M. Wang Q.. (2017). “Deformable image registration based on similarity-steered CNN regression,” in International Conference on Medical Image Computing and Computer-Assisted Intervention (Québec City, QC: Springer), 300–308. 10.1007/978-3-319-66182-7_35 PMC5731783 29250613

[B4] Chen L. Xiang Y. Chen Y. Zhang X. (2011). “Retinal image registration using bifurcation structures,” in IEEE International Conference on Image Processing (Brussels: IEEE), 2169–2172.

[B5] Chopra S. Hadsell R. LeCun Y. (2005). “Learning a similarity metric discriminatively, with application to face verification,” in 2005 IEEE Computer Society Conference on Computer Vision and Pattern Recognition (CVPR'05), Vol. 1 (San Diego, CA: IEEE), 539–546.

[B6] Cui Y. Zhou F. Lin Y. Belongie S. (2016). “Fine-grained categorization and dataset bootstrapping using deep metric learning with humans in the loop,” in Proceedings of the IEEE Conference on Computer Vision and Pattern Recognition (Las Vegas, NV: IEEE), 1153–1162.

[B7] Deng K. Tian J. Zheng J. Zhang X. Dai X. Xu M. (2010). Retinal fundus image registration via vascular structure graph matching. Int. J. Biomed. Imaging 2010, 906067. 10.1155/2010/906067 20871853 PMC2943092

[B8] Fischler M. A. Bolles R. C. (1981). Random sample consensus: a paradigm for model fitting with applications to image analysis and automated cartography. Commun. ACM 24, 381–395. 10.1145/358669.358692

[B9] Hadsell R. Chopra S. LeCun Y. (2006). “Dimensionality reduction by learning an invariant mapping,” in 2006 IEEE Computer Society Conference on Computer Vision and Pattern Recognition (CVPR'06), Vol. 2 (New York, NY: IEEE), 1735–1742.

[B10] He K. Zhang X. Ren S. Sun J. (2016). “Deep residual learning for image recognition,” in IEEE Conference on Computer Vision and Pattern Recognition (Las Vegas, NV: IEEE), 770–778.

[B11] Hernandez-Matas C. Zabulis X. Argyros A. A. (2020). Rempe: registration of retinal images through eye modelling and pose estimation. IEEE J. Biomed. Health Inform. 24, 3362–3373. 10.1109/JBHI.2020.2984483 32248134

[B12] Hernandez-Matas C. Zabulis X. Triantafyllou A. Anyfanti P. Douma S. Argyros A. A. (2017). Fire: fundus image registration dataset. Model. Artif. Intell. Ophthalmol. 1, 16–28. 10.35119/maio.v1i4.42

[B13] Hershey J. R. Chen Z. Le Roux J. Watanabe S. (2016). “Deep clustering: Discriminative embeddings for segmentation and separation,” in 2016 IEEE International Conference on Acoustics, Speech and Signal Processing (ICASSP) (Shanghai: IEEE), 31–35.

[B14] Hill D. L. Batchelor P. G. Holden M. Hawkes D. J. (2001). Medical image registration. Phys. Med. Biol. 46, R1. 10.1088/0031-9155/46/3/201 11277237

[B15] Huang C. Loy C. C. Tang X. (2016). “Local similarity-aware deep feature embedding,” in Advances in Neural Information Processing Systems, Vol. 29 (Barcelona).

[B16] Jie H. Shen L. Samuel A. Gang S. (2019). “Squeeze-and-excitation networks,” in IEEE Transactions on Pattern Analysis and Machine Intelligence (Salt Lake City, UT: IEEE). 10.1109/TPAMI.2019.2913372 31034408

[B17] Krebs J. Mansi T. Delingette H. Zhang L. Ghesu F. C. Miao S.. (2017). “Robust non-rigid registration through agent-based action learning,” in International Conference on Medical Image Computing and Computer-Assisted Intervention (Québec City, QC: Springer), 344–352.

[B18] Krizhevsky A. Sutskever I. Hinton G. E. (2012). “Imagenet classification with deep convolutional neural networks,” in NIPS (Nevada).

[B19] Law M. T. Urtasun R. Zemel R. S. (2017). “Deep spectral clustering learning,” in International Conference on Machine Learning (Lugano: PMLR), 1985–1994.

[B20] Li H. Fan Y. (2017). Non-rigid image registration using fully convolutional networks with deep self-supervision. arXiv preprint arXiv:1709.00799. 10.1109/ISBI.2018.8363757 30079127 PMC6070305

[B21] Liu J. He J. Qiao Y. Ren J. S. Li H. (2020). “Learning to predict context-adaptive convolution for semantic segmentation,” in European Conference on Computer Vision (Glasgow: Springer), 769–786.

[B22] Lowe D. G. (2004). Distinctive image features from scale-invariant keypoints. Int. J. Comput. Vis. 60, 91–110. 10.1023/B:VISI.0000029664.99615.94

[B23] Motta D. Casaca W. Paiva A. (2019). Vessel optimal transport for automated alignment of retinal fundus images. IEEE Trans. Image Process. 28, 6154–6168. 10.1109/TIP.2019.2925287 31283507

[B24] Movshovitz-Attias Y. Toshev A. Leung T. K. Ioffe S. Singh S. (2017). “No fuss distance metric learning using proxies,” in Proceedings of the IEEE International Conference on Computer Vision (Venice: IEEE), 360–368.

[B25] Oh Song H. Jegelka S. Rathod V. Murphy K. (2017). “Deep metric learning via facility location,” in Proceedings of the IEEE Conference on Computer Vision and Pattern Recognition (Honolulu, HI: IEEE), 5382–5390.

[B26] Oh Song H. Xiang Y. Jegelka S. Savarese S. (2016). “Deep metric learning via lifted structured feature embedding,” in Proceedings of the IEEE conference on computer vision and pattern recognition (Las Vegas, NV: IEEE), 4004–4012.

[B27] Oliveira F. P. Tavares J. M. R. (2014). Medical image registration: a review. Comput. Methods Biomech. Biomed. Eng. 17, 73–93. 10.1080/10255842.2012.670855 22435355

[B28] Opitz M. Waltner G. Possegger H. Bischof H. (2017). “Bier-boosting independent embeddings robustly,” in Proceedings of the IEEE International Conference on Computer Vision (Venice: IEEE), 5189–5198.

[B29] Paszke A. Gross S. Chintala S. Chanan G. Yang E. DeVito Z.. (2017). “Automatic differentiation in pytorch,” in ICLR.

[B30] Rivas-Villar D. Hervella Á. S. Rouco J. Novo J. (2022). Color fundus image registration using a learning-based domain-specific landmark detection methodology. Comput. Biol. Med. 140, 105101. 10.1016/j.compbiomed.2021.105101 34875412

[B31] Rohé M.-M. Datar M. Heimann T. Sermesant M. Pennec X. (2017). “Svf-net: learning deformable image registration using shape matching,” in International Conference on Medical Image Computing and Computer-Assisted Intervention (Québec City, QC: Springer), 266–274.

[B32] Ronneberger O. Fischer P. Brox T. (2015). U-Net: “Convolutional networks for biomedical image segmentation,” in *International Conference on Medical Image Computing and Computer-Assisted Intervention* (Munich).

[B33] Schroff F. Kalenichenko D. Philbin J. (2015). “Facenet: a unified embedding for face recognition and clustering,” in Proceedings of the IEEE Conference on Computer Vision and Pattern Recognition (Boston, MA: IEEE), 815–823.

[B34] Serradell E. Pinheiro M. A. Sznitman R. Kybic J. Moreno-Noguer F. Fua P. (2014). Non-rigid graph registration using active testing search. IEEE Trans. Pattern Anal. Mach. Intell. 37, 625–638. 10.1109/TPAMI.2014.2343235 26353266

[B35] Simonyan K. Zisserman A. (2014). “Very deep convolutional networks for large-scale image recognition,” in ICLR (Banff, AB).

[B36] Sohn K. (2016). “Improved deep metric learning with multi-class n-pair loss objective,” in Advances in Neural Information Processing Systems, Vol. 29 (Barcelona).

[B37] Sokooti H. Vos B.d Berendsen F. Lelieveldt B. P. Išgum I.. (2017). “Nonrigid image registration using multi-scale 3D convolutional neural networks,” in International Conference on Medical Image Computing and Computer-Assisted Intervention (Québec City, QC: Springer), 232–239.

[B38] Sotiras A. Davatzikos C. Paragios N. (2013). Deformable medical image registration: a survey. IEEE Trans. Med. Imaging 32, 1153–1190. 10.1109/TMI.2013.2265603 23739795 PMC3745275

[B39] Tsai C.-L. Li C.-Y. Yang G. Lin K.-S. (2009). The edge-driven dual-bootstrap iterative closest point algorithm for registration of multimodal fluorescein angiogram sequence. IEEE Trans. Med. Imaging 29, 636–649. 10.1109/TMI.2009.2030324 19709965

[B40] Vos B. D. D. Berendsen F. F. Viergever M. A. Staring M. Išgum I. (2017). “End-to-end unsupervised deformable image registration with a convolutional neural network,” in Deep Learning in Medical Image Analysis and Multimodal Learning for Clinical Decision Support (Québec City, QC: Springer), 204–212.

[B41] Wang J. Chen J. Xu H. Zhang S. Mei X. Huang J.. (2019). Gaussian field estimator with manifold regularization for retinal image registration. Signal Process. 157, 225–235. 10.1016/j.sigpro.2018.12.004

[B42] Wang J. Song Y. Leung T. Rosenberg C. Wang J. Philbin J.. (2014). “Learning fine-grained image similarity with deep ranking,” in Proceedings of the IEEE Conference on Computer Vision and Pattern Recognition (Columbus, OH: IEEE), 1386–1393.

[B43] Wang X. Hua Y. Kodirov E. Hu G. Garnier R. Robertson N. M. (2019). “Ranked list loss for deep metric learning,” in Proceedings of the IEEE/CVF Conference on Computer Vision and Pattern Recognition (Long Beach, CA: IEEE), 5207–5216.

[B44] Weinberger K. Q. Saul L. K. (2009). Distance metric learning for large margin nearest neighbor classification. J. Mach. Learn. Res. 10, 207–244.

[B45] Yang G. Stewart C. V. Sofka M. Tsai C.-L. (2007). Registration of challenging image pairs: initialization, estimation, and decision. IEEE Trans. Pattern Anal. Mach. Intell. 29, 1973–1989. 10.1109/TPAMI.2007.1116 17848778

[B46] Yang X. Kwitt R. Styner M. Niethammer M. (2017). Quicksilver: fast predictive image registration-a deep learning approach. Neuroimage 158, 378–396. 10.1016/j.neuroimage.2017.07.008 28705497 PMC6036629

[B47] Zhou J. Jin K. Gu R. Yan Y. Zhang Y. Sun Y.. (2022). Color fundus photograph registration based on feature and intensity for longitudinal evaluation of diabetic retinopathy progression. Front. Phys. 10, 978392. 10.3389/fphy.2022.978392

[B48] Zou B. He Z. Zhao R. Zhu C. Liao W. Li S. (2020). Non-rigid retinal image registration using an unsupervised structure-driven regression network. Neurocomputing 404, 14–25. 10.1016/j.neucom.2020.04.122

